# Supplementary figures and images for: Genetic Diversity and Evolution of Viruses Infecting Felis catus: A Global Perspective
Source: Viruses. 2023 Jun 7;15(6):1338. doi: 10.3390/v15061338 (PMC10301096; doi:10.3390/v15061338)

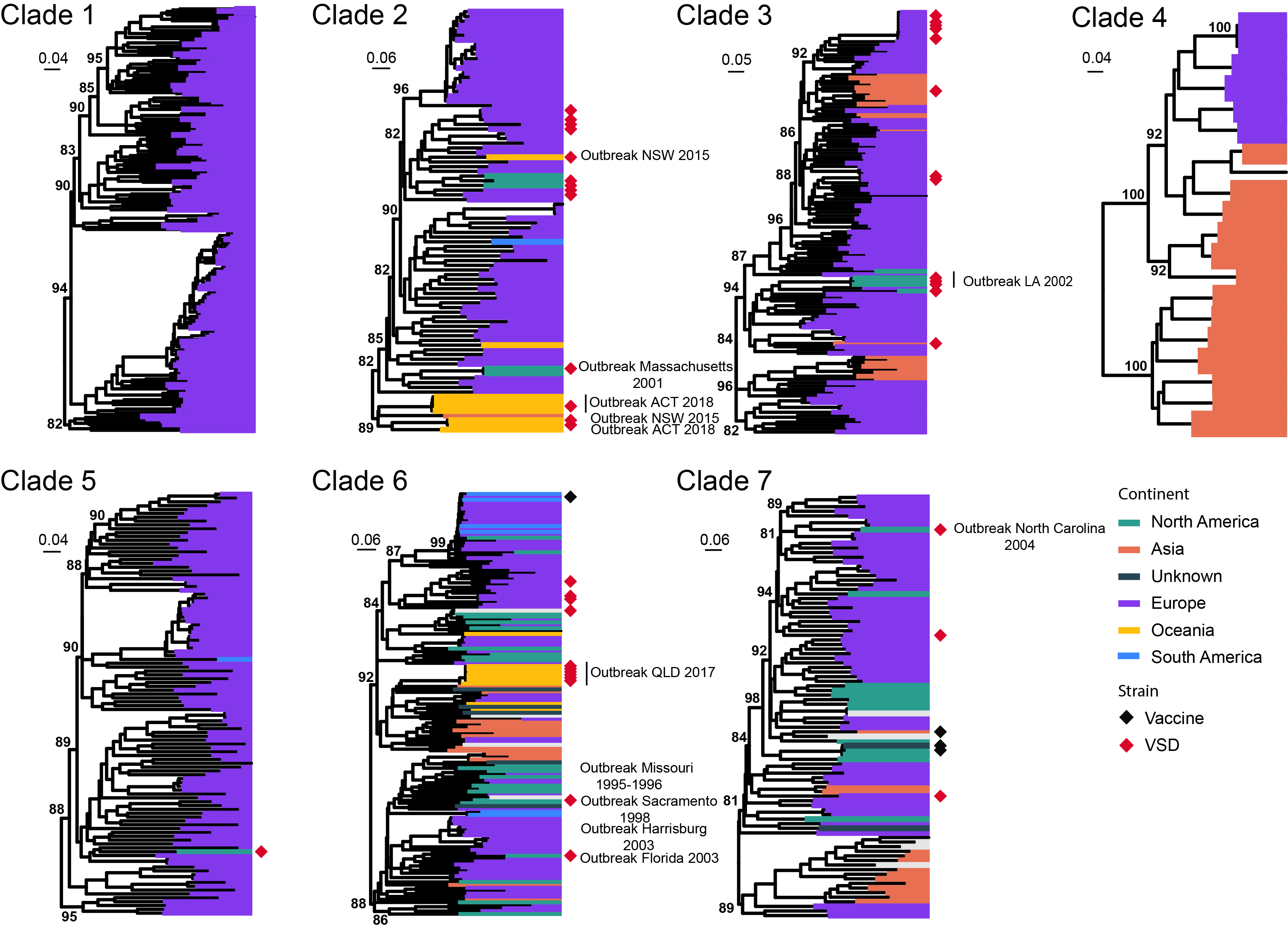

Supplement: Supplementary file 1 [file viruses-15-01338-s001.zip › Figure S1.jpg]

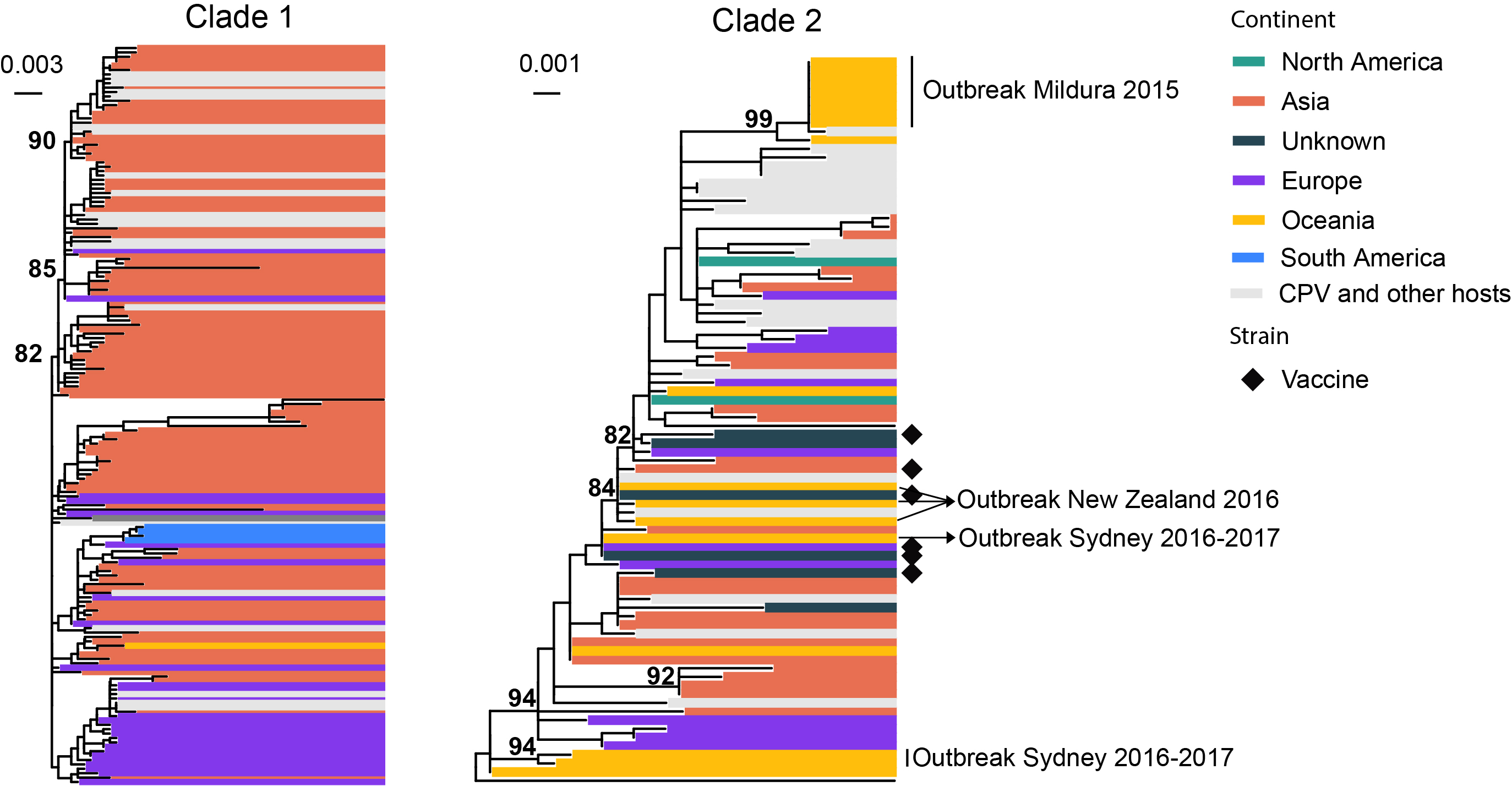

Supplement: Supplementary file 1 [file viruses-15-01338-s001.zip › Figure S2.jpg]
